# Detecting long-lived autodependency changes in a multivariate system via change point detection and regime switching models

**DOI:** 10.1038/s41598-018-33819-8

**Published:** 2018-10-23

**Authors:** Jedelyn Cabrieto, Janne Adolf, Francis Tuerlinckx, Peter Kuppens, Eva Ceulemans

**Affiliations:** Research Group of Quantitative Psychology and Individual Differences, KU Leuven – University of Leuven, Leuven, Belgium

## Abstract

Long-lived simultaneous changes in the autodependency of dynamic system variables characterize crucial events as epileptic seizures and volcanic eruptions and are expected to precede psychiatric conditions. To understand and predict such phenomena, methods are needed that detect such changes in multivariate time series. We put forward two methods: First, we propose KCP-AR, a novel adaptation of the general-purpose KCP (Kernel Change Point) method. Whereas KCP is implemented on the raw data and does not shed light on which parameter changed, KCP-AR is applied to the running autocorrelations, allowing to focus on changes in this parameter. Second, we revisit the regime switching AR(1) approach and propose to fit models wherein only the parameters capturing autodependency differ across the regimes. We perform a simulation study comparing both methods: KCP-AR outperforms regime switching AR(1) when variables are uncorrelated, while the latter is more reliable when multicolinearity is severe. Regime switching AR(1), however, may yield recurrent switches even when the change is long-lived. We discuss an application to psychopathology data where we investigate whether emotional inertia -the autodependency of affective states- changes before a relapse into depression.

## Introduction

In many scientific fields –ranging from financial economics and signal processing over ecology^1–9^ to emotion and abnormal psychology^10–14^–multivariate time series are collected to study how a system changes over time. Of the many features of a system that can be studied (e.g., mean, variance, correlation), a crucial one is its temporal dependency, reflecting how well system scores at specific time points can be predicted by scores at earlier time points. Temporal dependency is important as it provides insight into the system’s memory and predictability^1–3^ and even resilience^15,16^. For instance, emotional inertia, the tendency of feelings to carry over form one moment to the next, captures self-predictability of emotion and resistance to change, and has been found to be higher for individuals with low emotion-regulation skills^17^ and predictive for the development of depression^11,12^.

The simplest form of temporal dependency is autodependency, measuring the extent to which a variable can be predicted by itself at the previous moment. If the time intervals between observations are (almost) equally large, two statistical approaches can be employed to estimate autodependency. First, one can simply compute the autocorrelation, by calculating the correlation between a variable and its lagged version. The second approach is to fit an autoregressive lag one (AR(1)) model^1,2^, yielding a regression coefficient that quantifies autodependency (possibly on top of other model parameters). This coefficient will usually be almost equal to the autocorrelation.

Although most applications assume that the autocorrelation remains constant across time, there is ample evidence that this parameter may change as well across time. For instance, emotional inertia was demonstrated to change in response to triggers such as social stress^18^ or isolation^19^, and to increase before the onset of depression^11,12^. During sleep, the autocorrelation of EEG signals dramatically drops at the start of the REM phase^20^. The autocorrelation of seismic signals decreases abruptly during a volcanic eruption^21^. The onset of epileptic seizures is characterized by a strong increase in the autocorrelation of EEG scores^22,23^. So far, these changes have mostly been documented using knowledge about their timing (when did the volcano erupt or did a stressful event happen). However, this timing is often unknown. To predict crucial events and advance knowledge on the underpinnings of the phenomena of interest, methods that can reliably capture unknown autocorrelation changes are called for.

In this paper, we therefore aim to propose and evaluate two methods for screening multivariate time series for the presence of long-lived changes (i.e., changes that last for multiple successive time points) in the autocorrelation of at least one of the monitored variables: a non-parametric change point detection method and a regime switching AR(1) approach. Regarding the first, we will tailor KCP (Kernel Change Point)^24^, a general-purpose change point detection method that can signal changes in –in principle- any parameter, so that it focuses on autocorrelation changes. KCP locates change points by pooling subsequent time points that are characterized by similar distributions into a phase. To target autocorrelation changes, we propose to implement KCP to the running autocorrelations instead of the raw data. These running autocorrelations are derived by sliding a window across the time series and computing the autocorrelation in each window. The regime switching AR(1) approach^25,26^, allows the time series to recurrently switch between *R* different autodependency regimes. This switching is governed by a hidden Markov process that specifies the switching probabilities between any two regimes. Next to recurrent short-lived switches, the method can also capture longer-lasting changes, which is of interest in this paper.

While the performance of KCP and regime switching methods in detecting mean changes is well studied^27,28^, their performance in capturing changes in autocorrelations was not evaluated so far. In the next section, we will first demonstrate the key ideas of both methods through a toy example. Next, we report the results of an extensive simulation study, where the autocorrelation change may involve all or only a subset of the variables. In the simulations, we manipulated the size of the autocorrelation changes, the number of change points and the number of (noise) variables. We focus on evaluating the performance in terms of sensitivity (i.e., the power to detect autocorrelation changes) and deal with specificity (i.e., whether and how they are affected by changes in other parameters) in the discussion. We end by reanalyzing psychopathological data from Wichers *et al*.^12^. Plotting running autocorrelations, these authors found an increase in emotional inertia before the patient relapses to depression. We will confirm whether the data contain a significant autocorrelation change indeed, and if so, whether this change precedes depression relapse.

## Results

### Toy Example

**Figure 1:**
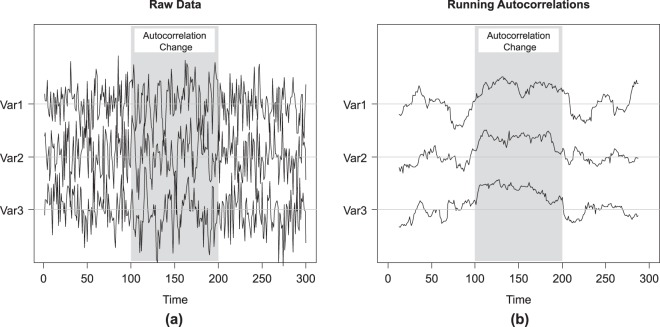
The raw scores (**a**) and running autocorrelations (**b**) for the toy example. A trivariate time series comprised of 300 time points was simulated from a multivariate normal distribution with zero means, unit variances and zero cross-correlations. Three phases are simulated with 100 time points each. In Phase 1, all autocorrelations are equal to zero. In Phase 2, a change was introduced such that all autocorrelations shifted to 0.50. In Phase 3, all autocorrelations reverted to zero. The different phases are indicated by the varying background shading.

We simulated a trivariate time series from a multivariate normal distribution with zero means, variances of one, and all correlations equal to zero. The time series consists of three phases of 100 time points each (Fig. 1a). In the first phase the variables have zero autocorrelations, in the second phase, an autocorrelation of 0.50 was introduced to all the variables, and in the third phase all autocorrelations become zero again. Because of the autocorrelation change in the second phase, we expect the variance to be somewhat higher in this phase.

#### KCP-AR (Kernel change point detection on the running autocorrelations)

**Figure 2:**
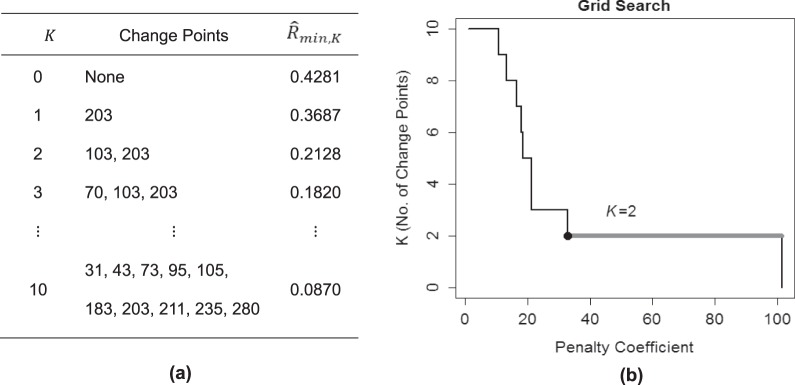
KCP-AR analysis for the toy example. In (**a**), the optimal change point locations and the within-phase variance, ${\hat{R}}_{min,K}$, for a given K are displayed. In (**b**), the grid search to find the most optimal K is exhibited.

KCP-AR detects autocorrelation changes, by implementing KCP, a non-parametric technique proposed by Arlot *et al*.^24^, on the running autocorrelations. Figure 1b shows the running autocorrelations for the toy example, computed within a window of size 25 that is slid across the time series. The time point that we associate with the autocorrelation computed in a window is the median of the time points included. Other possible choices can be made but this choice is optimal in case a change point is present as it would correspond to a window containing an equal number of time points from both phases, maximizing the chance of detecting it. To locate *K* change points, these running autocorrelations are pooled in *K* + 1 phases of subsequent values. These phases are as homogeneous as possible, as is quantified by a within-phase variance measure ${\hat{R}}_{min,K}$. This measure is based on pairwise similarities between the running autocorrelations in different windows, computed using the Gaussion Kernel function. In Section 4, a detailed description of the computation and optimization of ${\hat{R}}_{min,K}$ is provided. Figure 2a displays the ${\hat{R}}_{min,K}$-values obtained for the toy example letting *K* range from 0 to 10, and the corresponding optimal change point locations.

To decide on the adequate number of change points, KCP-AR employs two steps: In the first step, a significance test is conducted on the ${\hat{R}}_{min,K}$’s to decide whether the time series contains at least one significant autocorrelation change point. If this test is significant, we move on to step 2 and perform a grid search in which we linearly increment the penalty coefficient, *C*, proposed by Arlot *et al*.^24^ and retain the *K*-value that is most often selected across the considered *C*-values. Also, these steps will be elaborated in Section 4. For the toy example, a highly significant result was obtained in step 1 (p-value = 0), thus, we proceeded with the grid search (Fig. 2b) showing that the most frequently returned and therefore selected *K*-value is 2. Going back to the optimal change point locations in Fig. 2a, the corresponding change point locations are *T* = 103 and *T* = 203. KCP-AR, therefore, successfully located the two autocorrelation change points at *T* = 101 and *T* = 201, with a minimal delay of 2 time points.

#### Regime Switching AR(1) Model

Next, we fitted a regime switching AR(1) model to the toy data, using the dynr package^29^ and estimated 1 up to 3 regimes. The only model parameters that were allowed to change across the estimated regimes are the regression coefficients of the three variables. Thus, each observation, ***X***_*i*_, in the toy example was modelled as,

1 $${{\boldsymbol{X}}}_{i}={\boldsymbol{\alpha }}+{{\boldsymbol{\phi }}}^{r}{{\boldsymbol{X}}}_{i-1}+{{\boldsymbol{\varepsilon }}}_{i}$$

where ***α*** is the vector of intercepts, which we fixed to 0, ${{\boldsymbol{\phi }}}^{r}$ is the vector of regime dependent regression parameters, and ${{\boldsymbol{\varepsilon }}}_{{\boldsymbol{i}}}$ is the vector of innovations which are assumed to be independently sampled from a multivariate normal distribution with means equal to zero and covariance matrix, **∑**.

**Figure 3:**
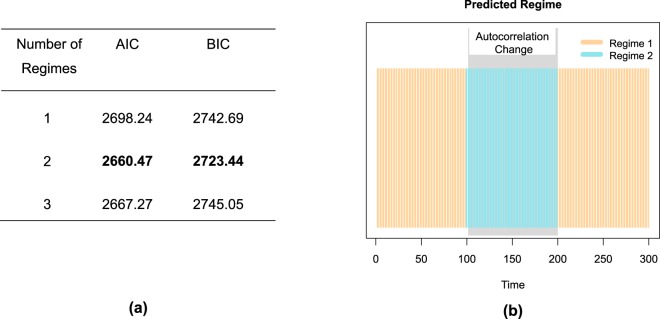
Regime switching AR(1) analysis for the toy example. In (**a**), the AIC and BIC values for the fitted models are tabulated, and in (**b**), the regime assignment for each time point based on the best model is displayed.

The optimal number of regimes can be decided on using the AIC and BIC values. As could be expected on how the data were generated, the model with two regimes yields the lowest AIC and BIC values (Fig. 3a) and should therefore be retained. To determine how well the method captures the location of the autocorrelation changes, we examined to which regimes the different time points are assigned. This assignment can be obtained by applying Bayes’ rule to the posterior probabilities with which a time point belongs to the different regimes (i.e., the time point is assigned to the regime with the highest posterior probability). Figure 3b exhibits these assignments for the toy example, showing that the majority of the time points at the beginning (until *T* = 98) and at the end of the time series (from *T* = 200) are classified into Regime 1, while those in the middle are assigned to Regime 2. Both changes are very proximal to the two real change points at *T* = 101 and *T* = 201. The regime switching AR(1) model with two regimes, therefore successfully recovered the abrupt autocorrelation changes we induced in the data. Note that it may happen, however, that the regime assignments exhibit very short-lived switches, which would suggest that no important longer-lasting changes occur.

### Simulation Study

The toy example demonstrated that both methods can detect autocorrelation change points. We now report the results of an extensive simulation study considering cases with one or two change points, to further compare their sensitivity. When monitoring a single system, variables are often dependent^30^, and when this collinearity is high, estimates are usually unstable (higher standard errors), resulting to lower power^31^. Thus, we also simulated both uncorrelated and correlated settings to investigate the robustness of the methods against this. At the end, we examined the false alarm rate by analyzing data in which the autocorrelation remains constant.

For the single change point case, two phases comprised of 100 time points each were simulated from a multivariate normal distribution. In the first phase, all autocorrelations are equal to zero, and in the second phase, a subset of the variables become autocorrelated. In the two change points case, three phases are simulated with 100 time points each. In the first and third phases all variable have zero autocorrelation, but in the second phase a subset of them become autocorrelated. We crossed the number of change points with the following factors and generated 100 replicates for each unique combination.

1. *Correlation between variables (fixed across all time points), r: 0, 0.60*
2. *Number of variables in the system, V*: 1, 2, 3, 5 and 7
3. *Number of changing variables S*: ranges from 1 (uncorrelated settings) or 2 (correlated settings) until *V* (the number of noise variables is V-S)
4. *Change in autocorrelation*, ${\rm{\Delta }}\phi$: *0.20, 0.40, 0.60*

Each simulated data set was analyzed with the regime switching AR(1) method, only allowing the regression parameters to switch, while the intercepts were set to zero and the covariance matrix was freely estimated but fixed across the time series. We always fitted 1, 2 and 3 regime models and employed AIC and BIC to choose the optimal number of regimes.

We also analyzed each data set with KCP-AR, using a window size of 25 to derive the running correlations. This choice was based on previous studies which revealed that this window size leads to more power and yields less biased KCP correlation change points in comparison to larger windows^32^. The number of change points was determined through the significance test and the grid search described above. These two steps require setting the maximum number of change points to be considered; we used 10.

Using the number of regimes indicated by AIC/BIC and the number of KCP change points suggested by the grid search, we computed the RI (Rand Index^33^) between the regime assignments (derived from applying Bayes’ rule on the posterior regime probabilities), the estimated KCP phases and the true phase memberships. The RI lies between 0 and 1, and the closer it is to 1, the more similar are the recovered and the underlying phases. Higher RI values, therefore, imply better recovery. Note that also in case the regime switches occur frequently, the RI score can be computed.

**Figure 4:**
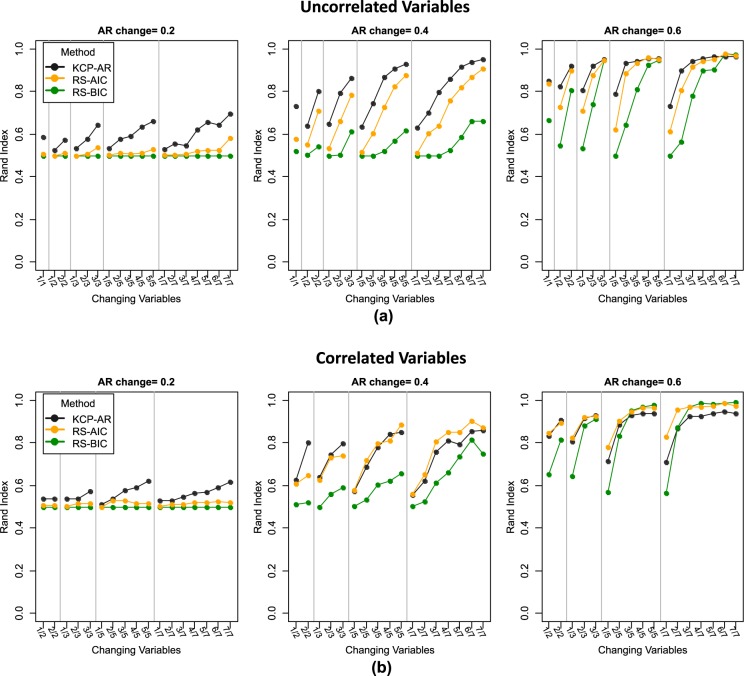
Rand Indices (RI’s) for the one change point case. RI’s for the uncorrelated and correlated settings are displayed in (**a**) and (**b**), respectively. KCP-AR is indicated in black, and the regime switching AR (1) method is in orange for AIC and in green for BIC. The settings in the x-axis are denoted as (no. of changing variables)/(total number of variables).

**Figure 5:**
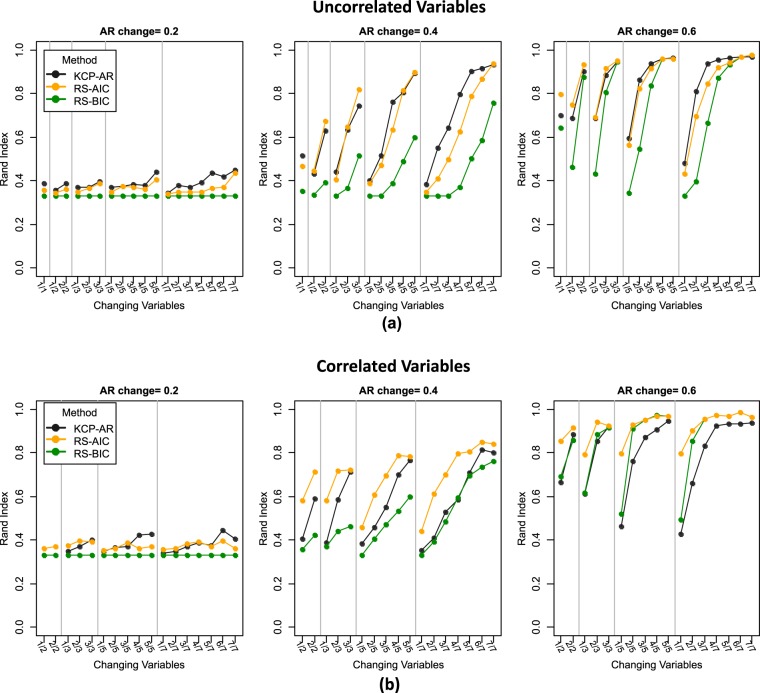
Rand Indices (RI’s) for the two change points case. RI’s for the uncorrelated and correlated settings are displayed in (**a**) and (**b**), respectively. KCP-AR is indicated in black, and the regime switching AR (1) method is in orange for AIC and in green for BIC. The settings in the x-axis are denoted as (no. of changing variables)/(total number of variables).

We focus first on the case where there is a single change point and the variables are uncorrelated (Fig. 4a). In this case, the performance of both methods is largely determined by the magnitude of the autocorrelation change. As we move from the left to the right panels, the change in autocorrelation becomes larger, and the RI’s obtained are higher. The RI values for a shift of 0.20 are very low, implying that detecting this minimal change is difficult, regardless of the method. As expected, the presence of noise variables strongly affects detection performance. KCP-AR was generally better than regime switching AR(1), especially yielding higher RI’s in difficult cases, where the autocorrelation change is minimal and/or noise variables outnumber the changing ones. For regime switching AR(1), AIC (in orange) proves better than BIC (in green) as it consistently yielded higher RI’s. Regarding the number of changes extracted, both methods often indicate that no change is present when the size of the autocorrelation change is small (Supplementary Fig. S1). When the size of the change increases, however, AIC retained three instead of two regimes in a considerable percentage of cases (i.e., across all conditions, the solution with three regimes was selected for 14% of the data sets on average, whereas this percentage went up to 42% in specific conditions). BIC tends to favor models with no change (one regime) for settings with many noise variables, yet it also yields too many regimes for a considerable number of data sets (i.e., up to 17% in specific conditions). KCP-AR, on the other hand, only seldom extracted too many change points (i.e., up to 7% in some settings). Most of the results in the one change point case extend to the two change points scenario (Fig. 5a, Supplementary Fig. S2), but the latter proved to be more difficult as the RI’s were generally lower.

When the variables are strongly correlated (corr = 0.60) throughout, an autocorrelation change of 0.20 remains difficult to detect (Fig. 4b). Except for the settings with 2 and 3 variables only, regime switching AR(1) outperforms KCP-AR, for both AIC and BIC. This result implies that KCP-AR’s detection performance is hampered by the correlation introduced between the variables. Specifically, KCP-AR has lower power than regime switching AR(1) since often no change points are extracted (Supplementary Fig. S3). As in the uncorrelated setting, the regime switching method (AIC) yields an extra third regime for a considerable number of data sets (up to 50% in some conditions), while BIC still performs worse, often choosing one regime in difficult settings yet also prone to overextraction (up to10%) when it declares a change. For KCP-AR, overextraction occurs only seldom (up to 4%). Results for settings with two change points are very similar (see Fig. 5b and Supplementary Fig. S4).

The simulation settings above were replicated, but this time, the phase sizes were reduced to 50 time points, to study how this reduction in information affects power. Results are very similar for the one change point case, where KCP-AR outperforms the regime switching method in the uncorrelated settings (Supplementary Fig. S5a), while the latter is more reliable when there is high collinearity between variables (Supplementary Fig. S5b). For the two change points case, the regime switching method (AIC) was clearly the better method, both in the uncorrelated (Supplementary Fig. S6a) and correlated (Supplementary Fig. S6b) settings. However, it should be emphasized that the power of both methods is only acceptable (≥0.80) for a very large autocorrelation change (0.60) or, for KCP-AR, if seven variables exhibited a 0.40 change (Supplementary Fig. S5a).

**Figure 6:**
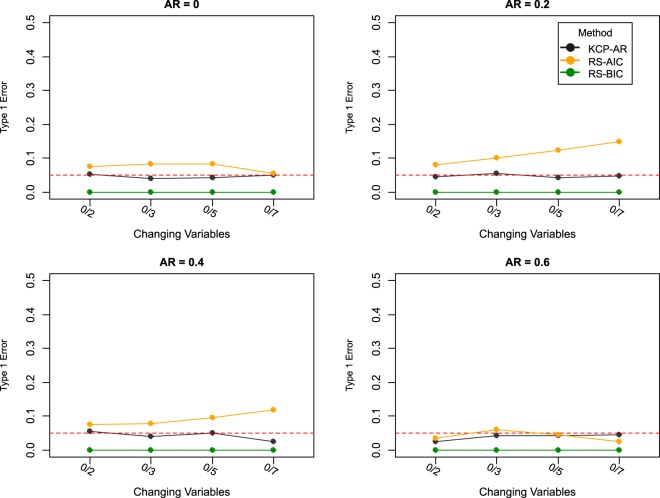
Type I error rate. The percentage of data sets for which false alarms were obtained are plotted for KCP-AR (in black) and the regime switching method (AIC – in orange; BIC in green). The x-axis indicates the settings which are written as (no. of changing variables)/(total number of variables).

Finally, we consider the results for data sets in which no autocorrelation change point is present to check how prone the methods are to false alarms. Type 1 error percentages were computed by counting the proportion of data sets for which the methods declared at least one change to be present (i.e., at least one change point for the case of KCP-AR and more than one regime for regime switching AR(1)). The same numbers of variables were considered, but this time, 500 replicates were simulated to get more reliable type 1 error percentages. Four autocorrelation levels were introduced to the time series and were kept constant all throughout (i.e., 0 and the autocorrelation levels tested above). In Fig. 6, it is evident that using BIC for regime switching AR(1) ensures a very conservative result when it comes to judging the evidence in favor of a change point. With KCP-AR, type 1 error rates are approximately around 0.05, which is the alpha value used in the KCP-AR significance test. Using AIC for the regime switching AR(1), on the other hand, leads to a higher chance of yielding false alarms with type 1 error rate reaching almost 15% in some settings.

### Illustrative example

We now turn to empirical data regarding relapse into depression. Examining a system of depression relevant mood states that are repeatedly measured for a single patient, we aim to confirm whether the onset of depression is preceded by a change in autocorrelation, as is predicted by theories on early warning signs for depression^11,34^. Wichers *et al*.^12^ analyzed the same time series, and indeed found evidence for elevated autocorrelations. This analysis was univariate and rather descriptive, however, in that all variables were aggregated into a single sum score, and the running autocorrelation of this sum score was monitored graphically to investigate whether it exhibited a rising trend. No further testing was done to determine the significance of the observed changes. The expected autocorrelation change in this data is rather gradual^11,34^. However, if this change is large enough and long-lived, the methods can in principle, detect this by segmenting the data into phases with different autocorrelation levels. The timing of the detected change, though, may come later relative to the start of the increasing trend, especially for KCP-AR, which needs a considerable number of time points in a phase to declare a change point. We are interested, therefore, to test whether a significant autocorrelation change can be detected by the methods before the depression relapse, thus predicting this critical event.

**Figure 7:**
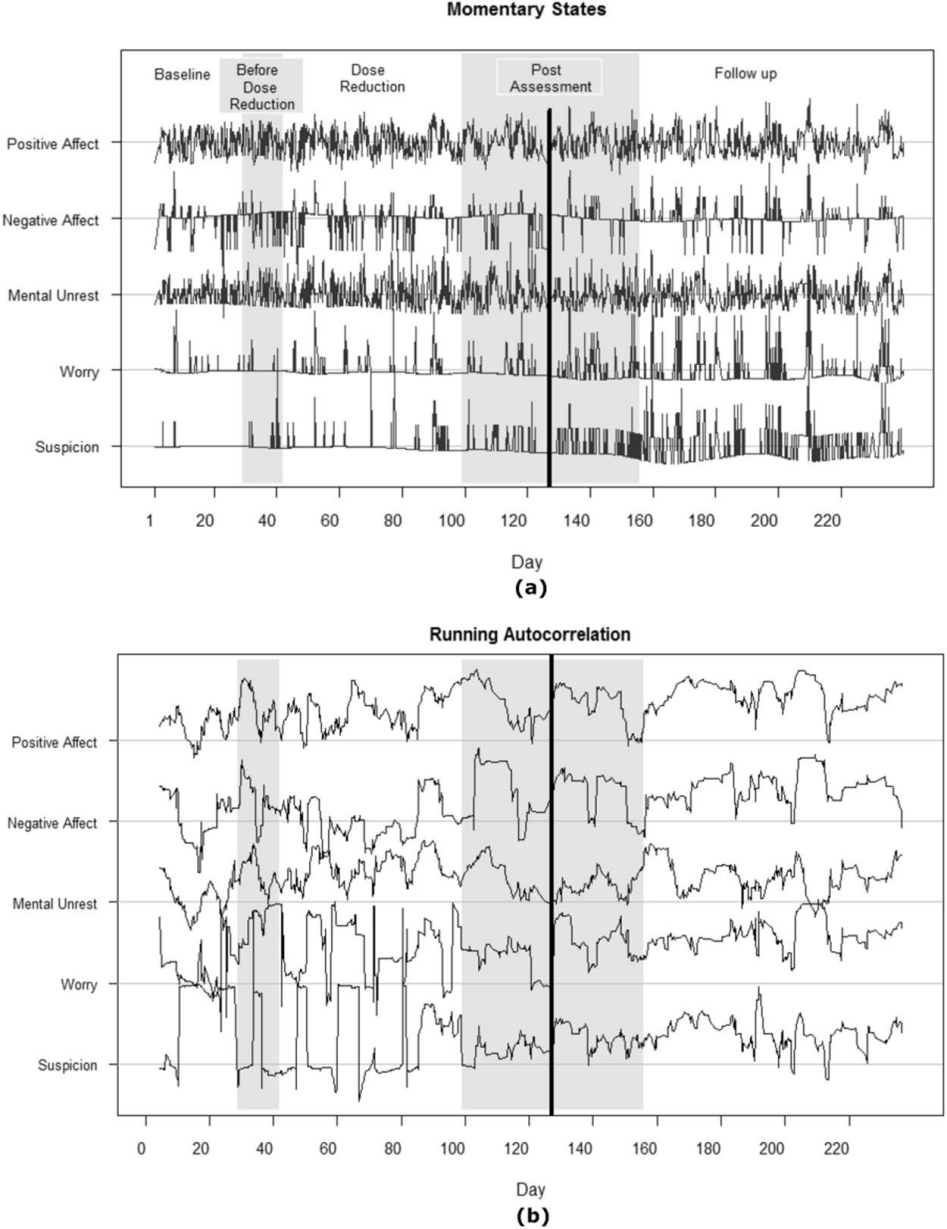
Momentary states (**a**) and their running autocorrelations (**b**). The experimental phases are indicated by the varying background shading, and the critical transition to the depressive state (Day 127) is marked by the black vertical line. Horizontal lines indicate zero autocorrelation for the corresponding variable. Range of running autocorrelations per variable: PA (−0.21, 0.88), NA (−0.64, 0.91), MU (−0.35, 0.77), Worry (−0.61, 0.99) and Suspicion (−0.46, 0.99).

The data is obtained from an experience sampling study of 239 days involving a participant treated for major depression, who agreed to undergo a dose reduction scheme of his anti-depressant. Each day of the study, the participant was beeped 10 times to report on his momentary experiences, generating multivariate data with1474 time points. Following Wichers *et al*.^12^ we will focus on five of these momentary experiences: negative affect, positive affect, mental unrest, worry and suspicion. The detrended time series is displayed in Fig. 7a, where the different experimental phases (i.e., baseline, double blind, post assessment and follow-up) are indicated by the varying background shading. To evaluate the depression status, depressive symptoms were monitored on a weekly basis using the SCL-90-R depression subscale. Using E-divisive^35^, another non-parametric and general-purpose change point detection method, Wichers *et al*.^12^ detected a critical transition point in the sum score of this subscale around Day 127 when the patient relapsed into depression. This critical transition point is indicated by the black vertical line in Fig. 7a.

Before implementing both methods, the data was pre-processed to ensure that the observations satisfy the AR(1) process. This was done by including only the time points for which lag 1 counterparts are available. Overnight lags were removed since the time intervals between these occasions are considerably larger. Moreover, we removed excessive outliers by replacing all scores exceeding the 3sd threshold with the maximum of the scores that stay within the threshold. This was done for each variable, separately.

#### KCP-AR Results

**Figure 8:**
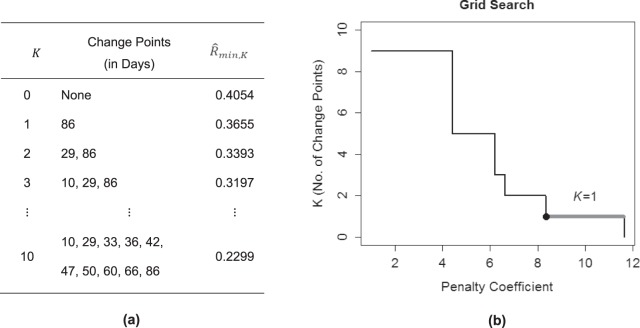
KCP-AR analysis: depression data. In (**a**), the optimal change point locations from zero up to ten change points and the corresponding within-phase variance, ${\hat{R}}_{min,K}$, are tabulated. In (**b**), the grid search is shown, suggesting that extracting one change point yields an adequate description of the data.

The running autocorrelations were obtained using a moving window of 25 time points (Fig. 7b). For *K* ranging from zero to ten, the location of the change points and the associated ${\hat{R}}_{min,K}$ –values are shown in Fig. 8a. Next, we tested whether there is at least one significant autocorrelation change point present in the data. Since the test yields a p-value of 0.01, we conclude that there is at least one autocorrelation change point present in the data.

Proceeding to the grid search, Fig. 8b shows that the most stable solution is *K* = 1, suggesting to retain one change point. The location of this single change point is at Day 86 (see Fig. 8a), which indeed precedes the known transition at Day 127. The dominant trend in the autocorrelation changes is that the autodependence becomes stronger after the change point. The most dramatic change was exhibited by suspicion (−0.04 to 0.38), followed by negative affect (0.14 to 0.42), positive affect (0.36 to 0.56) and worry (0.42 to 0.59). Mental unrest, on the other hand, barely changed in autocorrelation (0.39 to 0.36).

#### Regime Switching AR(1) Results

**Figure 9:**
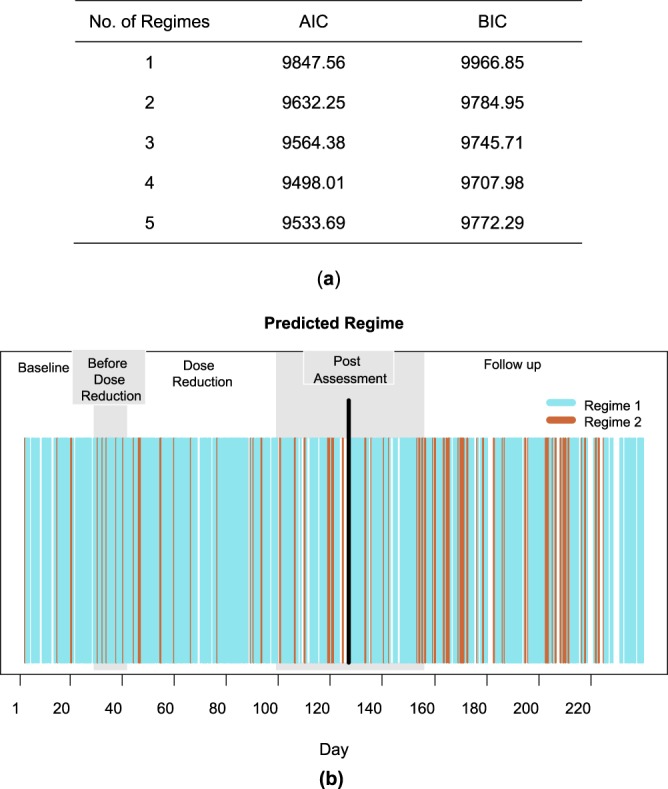
Regime switching AR(1) analysis: depression data. In (**a**), AIC and BIC values of the fitted regime switching AR(1) models are displayed. In (**b**), the assigned regimes under the 2 regime solution is exhibited. Each time point is classified into either the first (blue) or the second (brown) regime based on the posterior probabilities. The black vertical line indicates the critical transition to Depression at Day 127.

Regime switching AR(1) models were fitted using one up to five regimes, allowing only the autodependency parameters to vary per regime. In Fig. 9a, the model with four regimes yields the lowest AIC and BIC values. However, we retained the model with two regimes as both models lead to the same overall conclusions and the only significant difference between the parameters of the four- and two-regime models pertains to the regression parameter of Suspicion. Moreover, our simulation study shows that regime switching AR(1) models can be prone to overextraction.

In Fig. 9b, we display the regime to which each time point is assigned. The majority of the time points were classified into the first regime, but some short-lived switches to the second regime can be observed, especially after Day 153 which is already several days after the critical transition at Day 127. The first regime is characterized by absence of autodependence, as the regression parameters range from −0.06 to 0.12. In the second regime, regression parameters range from 0.65 to 0.93, implying strong autodependence. Since the switches to the second regime remain short-lived throughout, inferring a clear autocorrelation change point is not straightforward.

#### Summary

The results of both approaches do only partly concur. Both methods yield evidence that autodependency increases across time in this data. However, the regime switching AR(1) model reveals mostly short-lived changes in autocorrelation, which become more rampant only after the critical transition point, when the patient has already relapsed to depression. Using KCP-AR the conclusion aligns more with the running autocorrelations chart of Wichers *et al*.^12^, which suggest that the running autocorrelations rise before the known transition point.

## Discussion and Conclusion

In various application fields, crucial events are conjectured to be preceded or accompanied by long-lived autocorrelation changes. To verify these conjectures and understand the underpinnings of the phenomena under study, methods are needed that allow to screen time series for such changes. Yet, the available state-of-the-art methods, regime switching AR(1) models and KCP, are general-purpose, implying that they are also sensitive to other parameter changes including the mean, variance and correlations, and were mainly tested to detect such changes. This paper adds to the literature by showing how these methods can be tailored to focus on autocorrelation changes and by evaluating their performance in this regard.

Through the toy example, we have shown that regime switching AR(1) and KCP-AR can indeed detect abrupt autocorrelation changes. Our simulation results revealed that detection performance is influenced by the magnitude of the autocorrelation change, the number of change points, the number of noise variables, collinearity of the variables and the phase size. As expected, power increases for larger autocorrelation changes. It is worth noting that a small shift in autocorrelation (0.20) is extremely difficult to signal for both methods. When the variables are uncorrelated, KCP-AR is the more powerful method and also the most robust against noise variables. Collinearity of the variables, however, deteriorates KCP-AR’s performance, while that of regime switching AR(1) is enhanced. For KCP-AR, the power is probably lower when the variables are correlated because there is less independent information regarding the change in autocorrelation, obfuscating the permutation test. Regime switching AR(1), in contrast, includes covariance parameters dedicated to capture relationships between the variables. In the uncorrelated settings, estimating these parameters probably led to overfitting and yielded larger information criteria values. This led to a preference for the simpler yet incorrect model, including a single regime only. Lastly, our simulation settings consistently revealed that using AIC rather than BIC yielded better regime switching AR(1) results in terms of power. However, we stress that these results may depend on the length of the time series and the event. We expect BIC to perform better for longer time series.

For a shorter time series (phase size = 50), regime switching AR(1) is generally more sensitive than KCP-AR. This can be expected since KCP-AR employs a moving window technique, thus if the sample size is very small, the number of windows is even smaller, leading to less power. We, however, remind users that for both methods, acceptable power (≥0.80) can only be achieved if the autocorrelation change is very large (0.60), or in case of a smaller effect size (e.g., 0.40), when the number of variables exhibiting the autocorrelation change is at least 7. Such dramatic changes are rather unrealistic, thus we recommend to collect more than 50 time points per a priori expected phase. We have shown that, on top of the size of the change and the sample size, many factors can influence power (e.g., number of change points, collinearity between variables, number of variables). If prior knowledge on their expected values is available, we recommend to employ this to conduct a simulation study to assess and also optimize power for the considered experiment.

Turning to the illustrative example, both methods provided evidence for changes in autodependency, but there were also striking differences in the results, in that regime switching AR(1) did not yield a clear longer-lasting change but rather revealed some short-lived switches, whereas KCP-AR did yield one autocorrelation change. We remark that although the recovered changes for most of the variables are not very drastic (mean = 0.21, max = 0.43), the change point obtained was highly significant, which can be attributed to the abundance of time points for this specific data set (about 4 times longer than our one change point settings in the simulations). Going back to the differences in the obtained results, these naturally follow from the differences between the methods. As holds for all change point detection methods, KCP-AR can only locate longer-lasting change points that demarcate events occurring for a certain period^36^. The regime switching AR(1) method, on the other hand, can unravel both longer-lasting switches bounded by change points and short-lived switches. This means that we cannot rule out the possibility that the true pattern of autocorrelation changes might indeed be recurrent abrupt switching. At the end, this illustrative application nicely demonstrates how findings can differ when different statistical approaches are employed, and that users should be aware of this. We also recapitulate that in employing KCP-AR or regime switching AR(1), we do not impose that the change should be strictly abrupt and the goal is mainly to capture a long-lived autocorrelation change, which can predict relapse to depression. As a future direction, we also suggest that for this data, it certainly makes sense to also consider methods that can test for gradual autocorrelation changes^19,37^ as well as look for changes in other system features (e.g., correlation, as done by Cabrieto *et al*.^32,36^).

In this paper, our main goal was to evaluate the performance of the two approaches in terms of power, that is, their sensitivity to autocorrelation change. Future studies should investigate their specificity and inspect whether they can yield false detections that are driven by changes in other parameters such as the mean, variance and correlations. If this is the case, then these changes should be filtered out beforehand if the goal is to focus on locating autocorrelation change points. In our simulations, specificity is not an issue since only the autocorrelation parameter changes. In the illustrative data, mean changes were filtered out but variances and correlations can still change, and such changes might have influenced our results. For KCP-AR, we have initial evidence that such changes would not be picked up^38^, however. For regime switching AR(1), this remains to be tested. A solution would be to fit regime switching models in which also the variances and/or correlations are allowed to change across regimes, but further evaluation is needed to solve the associated model selection problem (which parameters should be free to vary) and to verify whether the correct timing and the correct parameters involved in the change can be effectively recovered. One plausible issue that may arise is that when mean changes co-occur with another, differently timed parameter change, change detection may be dominated by the mean change, leaving the other changes undetected^30^.

Because of the differences between both methods under study, we deem it necessary to enumerate both their strengths and limitations to guide users on deciding which method will better fit their data analytical needs. First, in terms of general detection performance, the two methods seem to perform comparably when the variables are uncorrelated. This is a very promising result since it implies that when the goal is detecting autocorrelation change points, two competitive methods coming from different statistical frameworks can be implemented. When variables are correlated, though, KCP-AR’s detection performance may be hampered. One way to address this is to implement dimension reduction techniques beforehand (PCA^39^) that can transform the variables into a few uncorrelated components. Future studies can investigate whether this will improve KCP-AR’s performance. We remark that this data-driven dimension reduction step is not implemented in the dynr package that we used to fit the regime switching AR(1) model. Extensions to state-space models, wherein the variables are mapped to latent variables, is possible, but the mapping should be known to the user a priori. We also note that both methods can be applied to each single variable separately as autodependency is essentially a univariate parameter. The advantage of such an approach would be that it allows the timing of the change point to differ across the variables. However, such an approach would be more prone to yield false alarms due to multiple testing and will also be less sensitive because the information provided by different variables is not integrated in the detection process.

Second, the methods differ in how easy it is to infer the location of the change point marking the long-lived change(s). KCP-AR automatically yields the change point estimate. When the change is large as in the toy example, inspecting the regime assignments derived from the regime switching AR(1) model can also yield straightforward change point estimates. However, as in the illustrative application, the method can also yield recurrent switches, complicating matters. One way to infer the change point in such cases is to slide a window across the time series and check at which window the shift to the next regime started to stabilize. With regards to quantifying the change occurring at a change point, in case of regime switching AR(1), one can look at the maximum likelihood estimates that are automatically generated. However, users should first scrutinize the number of regimes retained, as our simulation results revealed that even if information criteria are used to choose the best fitting model, the method can still be prone to overfitting, retaining too many regimes, and consequently, yielding too many parameters. For KCP-AR, on the other hand, auxiliary analysis is needed to compute and compare the autocorrelations per phase. This is reasonable since change point detection methods segment the series into phases with distinct parameter levels as in a piecewise constant function^24,40,41^. What sets the regime switching parameter estimates and the KCP-AR phase-specific autocorrelations apart is that they are based on different subsets of the data. Regime switching AR(1) considers all time points, while the estimates for KCP-AR are always computed within a phase. If there is an obvious change point, then we would expect these estimates to be similar, and those obtained with regime switching AR(1) can be preferred since they are estimated simultaneously (given that the AR(1) assumptions hold). However, if the regime switching method yields many short-lived switches rather than regimes that are clearly separated in time, it makes sense to look at the autocorrelations based on the KCP-AR change points.

Third, KCP-AR looks at the running autocorrelations whereas the regime switching model fits the original data. While computing these running scores KCP-AR should only take observations into account for which the preceding time point is available and the gaps between them are equal. In the illustrative example, we addressed this by choosing only the time points with lag 1 counterparts and by deleting the overnight lags. One may also consider imputation techniques^42,43^ to fill in the time series with plausible values. Regime switching AR(1), on the other hand, can naturally handle this unequal time interval issue by fitting a continuous-time AR(1) model^44–46^.

Finally, in terms of flexibility, both methods can be extended in many ways. Regime switching AR(1) models can be extended to handle state space models^26^, non-linear dynamics^47^, missing data and multiple subjects. In fact, the dynr package can accommodate all these extensions already^29^. KCP-AR, on the other hand, can be implemented in data sets for which prior information on the distribution is unknown. Next to autocorrelations, KCP can be adapted to signal changes in other statistics for which no change point detection techniques are developed yet such as the distance correlation (non-linear association between vectors^48^) or Jaccard index (similarity coefficient between two binary time series^49^).

We end by reminding readers that they can also consider alternative parametric approaches to detect autocorrelation change points, such as Auto-PARM^4^. The advantages of Auto-PARM are that, similar to KCP-AR, it automatically yields change points, and similar to regime switching AR(1), it immediately returns parameter estimates. Moreover, the method offers more flexibility as it does not impose a common lag order across the phases. It is therefore possible to obtain phases with different AR orders. However, Auto-PARM would generally require a way larger sample size to achieve an acceptable power. For instance, for an autocorrelation change of 0.40, the method would require almost 5 times more time points in comparison to KCP-AR and regime switching AR(1) to achieve sufficient power. We suggest that in applications where ample time points are available, or the expected autocorrelation change is large, this method be considered because of the mentioned advantages. We, however, emphasize that this method is also sensitive to other parameter changes, and currently, unlike KCP-AR and regime switching AR(1), the software is not designed to allow users to solely focus on detecting autocorrelation changes.

In conclusion, both KCP-AR and regime switching AR(1) can effectively capture long-lived autocorrelation changes. Our simulation results revealed that their overall performance in detecting such change points is largely comparable, but the latter may yield short-lived switches. KCP-AR performs better when variables are uncorrelated, while regime switching AR(1) is more reliable in case of strong collinearity. The regime switching method, being a full-blown modeling approach, comes with all the associated advantages such as maximum likelihood estimates for the model parameters and extensions to more complex models. KCP-AR, on the other hand, is an attractive alternative which does not impose many stringent distributional assumptions and the underlying ideas can in principle be used to detect change points in any type of summary statistic.

## Methods

### KCP-AR

#### Locating the change points

KCP locates change points by segmenting the time series into phases, such that the observations within each phase are as homogeneous as possible. This is carried out by examining the similarities between time points through a kernel function. In this paper, we employed the Gaussian kernel function, which is the most used kernel in the literature^50^. Furthermore, our previous studies^27,32,36^ revealed that it performs reliably in change point detection. Given a time series, ${\boldsymbol{X}}=\{{{\boldsymbol{X}}}_{1},{{\boldsymbol{X}}}_{2},\mathrm{...}{{\boldsymbol{X}}}_{n}\}$, where each observation ***X***_*i*_ represents a vector of scores on *V* variables, the Gaussian kernel based similarity between two time points, ***X***_*i*_ and ***X***_*j*_, is given by

2 $$Gk({{\boldsymbol{X}}}_{i},{{\boldsymbol{X}}}_{j}\,)=\exp \,(\frac{-\parallel {{\boldsymbol{X}}}_{{\boldsymbol{i}}}-{{\boldsymbol{X}}}_{j}{\parallel }^{2}}{2{h}^{2}}),$$

where the bandwidth, *h*, is obtained by taking the median Euclidean distance between all ***X***_*i*_’s and ***X***_*j*_’s. The similarity takes on values close to 1 if the scores are similar, while it approaches zero when they are distant. For KCP-AR, where the goal is to focus the detection on the autocorrelation, the raw data is first pre-processed to obtain a derived time series that reflect only the fluctuations in the statistic of interest. Specifically, we move a window, ***w***_*i*_, across the time series and compute the corresponding ***P***_*i*_, the vector of autocorrelations for the *V* variables in the system. KCP-AR thus looks at the similarity,

3 $$Gk({{\boldsymbol{P}}}_{i},{{\boldsymbol{P}}}_{j}\,)=\exp (\frac{-\parallel {{\boldsymbol{P}}}_{{\boldsymbol{i}}}-{{\boldsymbol{P}}}_{j}{\parallel }^{2}}{2{{h}_{P}}^{2}}),$$

and then computes the intra-phase scatter^51^,

4 $${\hat{V}}_{{\tau }_{1},{\tau }_{2},{\tau }_{3}\mathrm{...},{\tau }_{{\rm{K}}},m}=({\tau }_{m}-{\tau }_{m-1})-\frac{1}{{\tau }_{m}-{\tau }_{m-1}}\sum _{i={\tau }_{m-1}+1}^{{\tau }_{m}}\sum _{j={\tau }_{m-1}+1}^{{{\rm{\tau }}}_{m}}\,Gk({{\boldsymbol{P}}}_{i},{{\boldsymbol{P}}}_{j}\,),$$

where the indices, ${\tau }_{1},{\tau }_{2},\mathrm{...},{\tau }_{K}$, denote the locations of the phase boundaries, *m* is the current phase number, and ${\tau }_{m-1}+1$ and ${\tau }_{m}$ are the first and last time points of this phase. When the running autocorrelations included in the phase are more similar, the rightmost term of ${\hat{V}}_{m}$ becomes more negative, yielding a smaller intra-phase scatter.

To estimate the location of *K* change points, KCP-AR optimizes a variance criterion based on the sum of all *K* + 1 intra-phase scatters,

5 $$\hat{R}({\tau }_{1},{\tau }_{2},\mathrm{...},{\tau }_{K})=\frac{1}{w}\sum _{m=1}^{K+1}\,{\hat{V}}_{{\tau }_{1},{\tau }_{2},{\tau }_{3}\mathrm{...},{\tau }_{{\rm{K}}},m},$$

where *w* denotes the total number of windows. For instance, in the simplest case where a single change point is being estimated, the criterion $\hat{R}({\tau }_{1})$ is given by $\frac{1}{w}({\hat{V}}_{{\tau }_{1},1}+{\hat{V}}_{{\tau }_{1},2})$, where the two intra-phase scatters are measured given the phases delineated by ${\tau }_{1}$. KCP-AR tries all possible locations ${\tau }_{1}\in \{2,\,3,\mathrm{...},w-1\}$, and the time point ${\hat{\tau }}_{1}$ yielding the minimum $\hat{R}({\tau }_{1})$ determines the change point, ${\hat{\tau }}_{1}+1$. To locate multiple change points, the same procedure of minimizing the variance criterion is carried out such that the optimal change point locations, ${\hat{\tau }}_{1}+1$, ${\hat{\tau }}_{2}+1$, …${\hat{\tau }}_{K}+1$, are determined by

6 $${\hat{\tau }}_{1},{\hat{\tau }}_{2},\ldots ,{\hat{\tau }}_{K}={\rm{\arg }}\,{\rm{\min }}\,\hat{R}({\tau }_{1},\,{\tau }_{2},\mathrm{...},{\tau }_{K})$$

To simplify the notations as this will prove useful in describing the next KCP steps, we denoted the minimized variance criterion for every *K* as ${\hat{R}}_{min,K}$.

#### Choosing the number of change points

KCP can yield change points for any *K* specified by the user. However, it is not often the case that the number of change points is known a priori, implying that this number should be inferred from the data as well. Since the fit measure, ${\hat{R}}_{min,K}$, continues to decrease as more change points are introduced, a penalization strategy was proposed by Arlot *et al*.^24^ to choose a solution that balances the fit and complexity caused by adding more change points. The penalized term is given by,

7 $$\hat{K}={\rm{\arg }}\,{\rm{\min }}\,{\hat{R}}_{min,K}+pe{n}_{K},$$

where $pe{n}_{K}=C\frac{{V}_{max}(K+1)}{w}[1+\,{\rm{l}}{\rm{o}}{\rm{g}}(\frac{w}{K+1})]$. The penalty coefficient, *C*, determines the influence of the penalty term such that a larger *C*-value imposes a stronger penalty and would lead to less change points. The constant, *v*_*max*_, is estimated by taking the empirical covariance matrix of the first (and also the last) 5% of the time series and choosing whichever is the larger. This penalization scheme, though, strongly relies on the independence assumption, yielding excessive false alarms when KCP is applied to a highly dependent time series such as the running autocorrelations. To address this, Cabrieto *et al*.^36^ proposed to first conduct a test that confirms whether there is at least one change point in the running statistic, and if this test is significant, the most stable solution from the penalization scheme is determined through a grid search.

Significance test: The significance test we will employ is the variance drop test, which examines the improvement in the variance criterion caused by inducing an extra change point. The variance drop, ${\hat{R}}_{min,K}-\,{\hat{R}}_{min,K-1}$, is computed for all *K*’s, and the maximum drop is compared to that of the permuted data. If there is no change point in the data, introducing additional change points will not yield significant improvement in the variance. However, if there is strong evidence for a change point then the fit improvement for the original data will be significantly larger than that of reshuffled data. Thus, we compare the maximal ${\hat{R}}_{min,K}-{\hat{R}}_{min,K-1}$ to the distribution obtained from reshuffling the data for a large number of times. In this paper, we always set the significance level to 0.05, implying that the permutation test is significant once the maximal ${\hat{R}}_{min,K}-{\hat{R}}_{min,K-1}$ exceeds the 95th percentile cut-off of the permuted distribution. We note that Cabrieto *et al*.^36^ proposed two sub-tests (i.e., the variance and the variance drop tests) for testing the presence of change points in the running statistics but we only employed the variance drop test since the variance test on running autocorrelations would lead to an inflated Type 1 error in cases when there is no change point but the autocorrelation is high.

Grid Search: Once the permutation based test above is significant, the grid search is performed. Going back to the penalized term in Equation (7), the choice of the penalty coefficient, *C*, directly influences the retained number of change points, *K*. Thus, the grid search aims to determine which *K* is optimal without relying too much on the choice of *C*. Hence, a range of *C*-values is plugged into the algorithm and the most stable *K* is identified. It proceeds by first setting *C* equal to 1, which will always lead to $K={K}_{max}$. Then, *C* is increased linearly by a certain increment, strengthening the influence of the penalty term. The algorithm, thus, returns decreasing *K*-values as *C* is increased. The grid search terminates when *C* becomes too large, yielding *K* = 0. The retained *K* is the most stable solution, which is the most frequently returned *K* during the grid search.

### Regime Switching AR(1) Model

The regime switching AR(1) model for a time series, ${\boldsymbol{X}}=\{{{\boldsymbol{X}}}_{1},{{\boldsymbol{X}}}_{2},\mathrm{...}{{\boldsymbol{X}}}_{n}\}$, where each observation ***X***_*i*_ represents a vector of scores on *V* variables, is written as

8 $${{\boldsymbol{X}}}_{i}={{\boldsymbol{\alpha }}}^{r}+{{\boldsymbol{\phi }}}^{r}{{\boldsymbol{X}}}_{i-1}+{{\boldsymbol{\varepsilon }}}_{i},$$

where the regime dependent parameters include, ***α***^*r*^, the vector of intercepts, ${{\boldsymbol{\phi }}}^{r}$, the vector of regression parameters describing the dependence between two successive time points, and ${{\boldsymbol{\varepsilon }}}_{i}$, the vector of independent and identically distributed residuals drawn from a multivariate normal distribution with means equal to zero and also a regime dependent covariance matrix, ${{\boldsymbol{\Sigma }}}^{r}$. Given *R* regimes, the hidden Markov process that governs the switching between any two regimes is characterized by transition probabilities,

$$[\begin{array}{cccc}{p}_{11} & {p}_{12} & \cdots  & {p}_{1R}\\ {p}_{21} & {p}_{22} & \cdots  & {p}_{2R}\\ \vdots  & \vdots  & \ddots  & \vdots \\ {p}_{R1} & {p}_{R2} & \cdots  & {p}_{RR}\end{array}]$$

where *p*_*st*_ is the probability of switching from regime *s* to regime *t*, and *p*_*ss*_ is the probability of staying in regime *s*. In this paper we used the dynr package to fit the regime switching AR(1) model. This package employs the algorithm proposed by Kim and Nelson^26^, which consists of five general steps^28,52^. The first three resemble the Kalman filter steps that yield a predicted value for every ***X***_*i*_. The last two steps are the approximation and collapsing steps which are necessary for the regime switching extension. Denoting the observation ***X***_*i*_ which implies a switch from regime *s* to *t* as ${{\boldsymbol{X}}}_{i}^{st}$, we enumerate these steps below:

1. *Prediction step*. First, the predicted value of ${{\boldsymbol{X}}}_{i}^{st}$ is estimated based on the information from all preceding time points, ${{\boldsymbol{X}}}_{i-1|i-1}^{s}$. This prediction is denoted as ${{\boldsymbol{X}}}_{i|i-1}^{st}$.
2. *One-step-ahead prediction error*. Then, the actual observation ***X***_*i*_ is compared to the prediction, ${{\boldsymbol{X}}}_{i|i-1}^{st}\,$resulting from step 1. Specifically, the one-step-ahead-prediction-error, ${{\boldsymbol{d}}}_{i}^{(s,t)}$, is the difference between these two values.
3. *Update step*. The prediction is updated obtaining ${{\boldsymbol{X}}}_{i|i}$, which takes into account the actual observation, ***X***_*i*_, and the one-step-ahead-prediction error, ${{\boldsymbol{d}}}_{i}^{(s,t)}$.
4. *Approximation step*. For steps 1 to 3, it is assumed that both *s* and *t* are known, but to yield the predictions, one would have to consider *R* switching possibilities per time point, yielding *R*^*n*^ trajectories for a time series with *n* time points. This can quickly become computationally intractable; therefore Kim^53^ proposed an approximation, which requires the quantity, ${\rm{P}}[{r}_{i-1}=s|{r}_{i}=t,{{\boldsymbol{X}}}_{1:i}]$, denoting the probability that the system previously belonged to regime *s* while it is now in regime *t* given all observed data until time point *i*, ***X***_1:__*i*_. This probability is estimated using the Hamilton filter^2,53^ for all combinations of *s* and *t*.
5. *Collapsing step*. Finally, a collapsing step is carried out on the probability estimates from step 4 to estimate ${{\boldsymbol{X}}}_{i|i}^{s}$, which will be used in step 1 when predicting the next time point.

To estimate the parameters of this model, the following log likelihood function is optimized,

9 $$log\,L=\sum _{i=1}^{n}\,log\,f({{\boldsymbol{X}}}_{i}|{{\boldsymbol{X}}}_{1:i-1})$$

where $f({{\boldsymbol{X}}}_{i}|{{\boldsymbol{X}}}_{1:i-1})$ is the likelihood of ***X***_*i*_, given all observed preceding data. This likelihood can be written as,

10 $$f({{\boldsymbol{X}}}_{i}|{{\boldsymbol{X}}}_{1:i-1})=\sum _{s=1}^{R}\sum _{t=1}^{R}\,f({{\boldsymbol{X}}}_{i}|{r}_{i-1}=s,\,{r}_{i}=t,{{\boldsymbol{X}}}_{1:i-1})\,{\rm{P}}[{r}_{i-1}=s,\,{r}_{i}=t,|{{\boldsymbol{X}}}_{1:i-1}]$$

with

11 $$f({{\boldsymbol{X}}}_{i}|{r}_{i-1}=s,\,{r}_{i}=t,{{\boldsymbol{X}}}_{1:i-1})={(2\pi )}^{V/2}|{{\boldsymbol{\Sigma }}}^{(t)}{|}^{-1/2}exp\{-\frac{1}{2}{{\boldsymbol{d}}}_{i}^{(s,t)\text{'}}{{\boldsymbol{\Sigma }}}^{({\boldsymbol{t}})-1}\,{{\boldsymbol{d}}}_{i}^{(s,t)}\}.$$

In this paper, we focused on evaluating the power to capture pure autocorrelation changes. Thus, we fitted models that only allowed for changes in the parameters in ${\boldsymbol{\phi }}$.

## Electronic supplementary material

Supplementary Figures

## Data Availability

All codes used are available upon request from the corresponding author. The depression data is publicly available at https://openpsychologydata.metajnl.com/articles/10.5334/jopd.29/.
