# Supplementary material for: Detecting long-lived autodependency changes in a multivariate system via change point detection and regime switching models
Source: Sci Rep. 2018 Oct 23;8:15637. doi: 10.1038/s41598-018-33819-8 (PMC6199340; doi:10.1038/s41598-018-33819-8)
Supplement: Supplementary file 1 — Supplementary Figures [file 41598_2018_33819_MOESM1_ESM.docx]

Detecting long-lived autodependency changes in a multivariate system via change point detection and regime switching models.

Jedelyn Cabrieto^1*^, Janne Adolf^1^, Francis Tuerlinckx^1^, Peter Kuppens^1^ and Eva Ceulemans^1^

^1^[Research Group of Quantitative Psychology and Individual Differences](https://ppw.kuleuven.be/okp/),

KU Leuven – University of Leuven, Belgium

| **KCP-AR**  ****  **(a)** |
| --- |
| **Regime Switching AR(1) - *AIC*  **  **(b)** |
| **Regime Switching AR(1) - *BIC***  ****  **(c)** |
| *Figure S1.* Number of recovered change points (or regimes) for the **one** change point case - **uncorrelated** settings. In **(a)**-**(c), t**he distribution of the number of recovered regimes for KCP-AR, regime switching method (AIC) and regime switching method (BIC) are exhibited, respectively. Detailed legends are provided in the leftmost panels (blue-green bars indicate correct recovery). |

| **KCP-AR**  ****  **(a)** |
| --- |
| **Regime Switching AR(1) - *AIC ***  **(b)** |
| **Regime Switching AR(1) - *BIC***  ****  **(c)** |
| *Figure S2.* Number of recovered change points (or regimes) for the **two** change point case - **uncorrelated** settings. In **(a)**-**(c), t**he distribution of the number of recovered regimes for KCP-AR, regime switching method (AIC) and regime switching method (BIC) are exhibited, respectively. Detailed legends are provided in the leftmost panels (blue-green bars indicate correct recovery). |

| **KCP-AR**  ****  **(a)** |
| --- |
| **Regime Switching AR(1) - *AIC*  **  **(b)** |
| **Regime Switching AR(1) - *BIC***  ****  **(c)** |
| *Figure S3.* Number of recovered change points (or regimes) for the **one** change point case - **correlated** settings. In **(a)**-**(c), t**he distribution of the number of recovered regimes for KCP-AR, regime switching method (AIC) and regime switching method (BIC) are exhibited, respectively. Detailed legends are provided in the leftmost panels (blue-green bars indicate correct recovery). |

| **KCP-AR**  ****  **(a)** |
| --- |
| **Regime Switching AR(1) - *AIC*  **  **(b)** |
| **Regime Switching AR(1) - *BIC ***  **(c)** |
| *Figure S4.* Number of recovered change points (or regimes) for the **two** change point case - **correlated** settings. In **(a)**-**(c), t**he distribution of the number of recovered regimes for KCP-AR, regime switching method (AIC) and regime switching method (BIC) are exhibited, respectively. Detailed legends are provided in the leftmost panels (blue-green bars indicate correct recovery). |

| **Uncorrelated Variables**  ****  **(a)** |
| --- |
| **Correlated Variables**  ****  **(b)** |
| *Figure S5.* Rand Indices (RI’s) for the **one change point** case (phase size=50). RI’s for the uncorrelated and correlated settings are displayed in **(a)** and **(b)**, respectively. KCP-AR is indicated in black, and the regime switching AR (1) method is in orange for AIC and in green for BIC. The settings in the x-axis are denoted as (no. of changing variables)/(total number of variables). |

| **Uncorrelated Variables**  ****  **(a)** |
| --- |
| **Correlated Variables**  ****  **(b)** |
| *Figure S6.* Rand Indices (RI’s) for the **two change point** case (phase size=50). RI’s for the uncorrelated and correlated settings are displayed in **(a)** and **(b)**, respectively. KCP-AR is indicated in black, and the regime switching AR (1) method is in orange for AIC and in green for BIC. The settings in the x-axis are denoted as (no. of changing variables)/(total number of variables). |
